# Colonic cytomegalovirus detection by mucosal PCR and antiviral therapy in ulcerative colitis

**DOI:** 10.1371/journal.pone.0183951

**Published:** 2017-09-08

**Authors:** Koki Okahara, Naoyoshi Nagata, Takayuki Shimada, Akane Joya, Tsunefusa Hayashida, Hiroyuki Gatanaga, Shinichi Oka, Toshiyuki Sakurai, Naomi Uemura, Junichi Akiyama

**Affiliations:** 1 Department of Gastroenterology and Hepatology, National Center for Global Health and Medicine, Tokyo, Japan; 2 Department of Gastroenterology and Hepatology, Juntendo Hospital, Tokyo, Japan; 3 Department of AIDS Clinical Center, National Center for Global Health and Medicine, Tokyo, Japan; 4 Department of Gastroenterology and Hepatology, National Center for Global Health and Medicine, Kohnodai Hospital, Chiba, Japan; University of San Francisco, UNITED STATES

## Abstract

**Background:**

We aimed to identify the risk factors associated with colonic cytomegalovirus (CMV) infection in ulcerative colitis (UC) and to compare the clinical course between antiviral therapy-treated and -untreated groups in mucosal CMV-polymerase chain reaction (PCR) -positive cases.

**Methods:**

We retrospectively selected 46 UC patients (>15 years old) in active phase who underwent colonoscopy with biopsy and were analyzed for CMV infection by mucosal PCR between October 2011 and December 2015 at our institution. Colonic CMV in inflamed mucosa was detected using quantitative real-time PCR. The clinical course was evaluated, including need for drug therapy/surgery or drug therapy intensification. In addition, we evaluated the clinical course between CMV-DNA− cases and CMV-DNA+ cases with low viral load.

**Results:**

At baseline, CMV-DNA+ patients were significantly older, had higher endoscopic scores, and required higher corticosteroid doses during the past 4 weeks than CMV-DNA− patients (*p*< 0.05). No significant differences were observed in disease duration, disease distribution, laboratory data, or use of other medication between CMV-DNA+ and CMV-DNA− patients. In the anti-CMV-treated group with a median (range) DNA load of 16,000 (9,000–36,400), 3patients achieved remission without additional UC therapy, 2 required additional UC therapy, and 1 required colectomy despite azathioprine and infliximab therapy. In the CMV-untreated group with a median (range) DNA load of 919 (157–5,480), all patients achieved remission with UC therapy alone. No significant difference was observed in the clinical course between CMV-DNA− cases and CMV-DNA+ cases with low viral loads.

**Conclusions:**

Aging, endoscopic UC activity, and corticosteroid dose predispose to colonic CMV infection, as determined by mucosal PCR, in UC. UC treatment without anti-CMV therapy may be warranted, particularly in patients with low-load CMV-DNA. Anti-CMV therapy alone does not always achieve clinical response in UC even in cases with high-load PCR.

## Introduction

Cytomegalovirus (CMV) infection has been frequently detected in ulcerative colitis (UC) patients[1]. UC patients are not only immunosuppressed due to immunosuppressive therapy but are often in a catabolic state and possibly have impaired natural killer T-cell function[2]. However, the role of CMV infection in UC patients remains unclear and previous data are conflicting as to whether CMV worsens inflammation in patients with severe colitis or is merely a marker of severe disease[3–7]. Previous studies have shown that detection of CMV in inflamed intestinal tissue predicts resistance to immunosuppressive therapy or the need for colectomy in UC[5,6,8], which supports a role for CMV infection as being “pathogenic” in UC exacerbation. Conversely, other studies have shown that UC patients have gone into remission with conventional immunosuppressive therapies alone or with intensive granulocyte and monocyte adsorptive apheresis without anti-CMV therapy[4,7], which suggests that CMV in UC is just an “innocent bystander”. If CMV infection is “pathogenic” in UC exacerbation, then anti-CMV therapy is indicated; however, the effectiveness of anti-CMV therapy in CMV+ patients has not yet been determined[9]. In particular, very little data are available on cases that are not treated with anti-CMV agents[6].

The European Crohn’s & Colitis Organization (ECCO) guideline for diagnosing CMV in colonic mucosa recommends detecting CMV DNA in tissue biopsies through polymerase chain reaction (PCR) analysis. The specific objectives of this study were to elucidate what clinical factors are associated with colonic CMV detection by mucosal PCR in UC patients and to examine the difference of clinical course of UC between an anti-CMV-treated group and an anti-CMV-untreated group in CMV-DNA+ cases.

## Materials and methods

### Study design, setting, and participants

We retrospectively selected 46 UC patients (>15 years old) in active phase who underwent colonoscopy with biopsy and were evaluated for CMV infection by mucosal DNA between October 2011 and December 2015 at our institution. The diagnosis of UC was based on clinical, endoscopic, radiological, and histological findings[6]. Steroid-refractory UC is defined as persistent acute symptomatic disease despite steroid therapy or as chronically active disease requiring continuous treatment for relief of symptoms[10]. Conversely, steroid-dependent UC is defined as inability to lower prednisolone dose to 10 mg/day to keep IBD inactive for 3 months, or as relapse within 3 months or less after suspending corticosteroid treatment[11]. According to our specific institutional protocol, we distinguished UC from infectious colitis by whether fecal, mucosal, and bacterial culture yielded no specific pathogens on the day of endoscopy[12]. We tested for several pathogenic bacteria including *clostridium difficile*, *salmonella* species, *campylobact*er *jejuni*, *entamoebahistolytica*, and *mycobacterium tuberculosis*, and this served to differentiate infectious colitis from IBD [12]. This study was conducted according to the principles of the Declaration of Helsinki and was approved by the ethics committee of National Center for Global Health and Medicine (approval date, August 5, 2016; approval No.814). The need for patient consent was waived because patient information was anonymized and identified before analysis.

### Clinical findings

We evaluated clinical variables including duration of disease, general status of patients, involvement distribution, laboratory findings, and medication history by an electronic medical database (MegaOak, NEC, Tokyo, Japan) on the day of endoscopy. We classified duration of disease as <1 year, 1–5 years, and > 5years. General status was assessed using the disease activity index score (0–12) as follows: stool frequency, rectal bleeding, endoscopic findings, and physician global assessment[13]. We classified involvement distribution into two groups: extensive colitis and left-sided colitis[14]. Treatment included5-aminosalicylic acid (ASA), corticosteroids, azathioprine, apheresis, tacrolimus, cyclosporine, infliximab, and adalimumab. The dose of corticosteroids was defined as the total dose given in 4weeks before endoscopy, as previously reported[7]. Clinical response was defined as improvement of clinical presentation and/or endoscopic remission.

### CMV-antigenemia and CMV pathological findings

The pp65 CMV-antigenemia assay using C10/C11 monoclonal antibodies (Mitsubishi Chemical Medience, Tokyo, Japan) was performed as described previously[15]. A positive result for the CMV-antigenemia assay was defined as ≥1 CMV-positive cell per 300,000 granulocytes applied. Biopsy specimens were subjected to staining with hematoxylin and eosin and immunohistochemical (IHC) analysis with anti-CMV monoclonal antibodies[16]. Results were considered positive when the mentioned cells showed marked brown coloration in both nuclei and cytoplasm[15].

### DNA extraction and PCR for CMV-DNA

Tissue samples were obtained during colonoscopy and were stored at −80°C until processing for DNA isolation. DNA for PCR assay was extracted from inflamed colonic mucosa obtained from patients at endoscopic examination using QIAamp DNA Mini Kit (Qiagen, Tokyo, Japan) in accordance with the manufacturer’s instructions. Quantitative real-time PCR was performed to detect CMV by using TaqMan Universal PCR Master Mix (Thermo Fisher Scientific, Kanagawa, Japan), previously published sets of primers and probes[17], and 7900HT Fast Real Time PCR System (Thermo Fisher Scientific) with the following thermal cycles; first at 50°C for 2 min and 95°C for 10 min, then 50 cycles of [95°C for 15 s, 60°C for 60 s]. Human CMV (HCMV, AD169 strain) Quantitated Viral DNA (Advanced Biotechnologies Inc., Eldersburg, MD) was used as a standard for CMV-DNA quantification. Input DNA was 50 ng and the detection limit was 4 copies/ 50 ng. The positive controls of each viral DNA were purchased from Advanced Biotechnologies Inc.[17].

### Statistical analysis

Firstly, we evaluated the baseline characteristics associated with CMV-DNA+ patients. Then, we examined the clinical course after the initial endoscopy date during the hospital stay. In CMV-DNA+ patients, we examined the baseline characteristics of UC, CMV characteristics, and UC treatment and clinical course between patients who received anti-CMV therapy and those who did not. Clinical course was classified as clinical response with (i) only 5-ASA treatment, (ii) additional steroid therapy alone, (iii) additional steroid therapy and immunosuppressant/immunomodulator therapy, and (iv) additional steroid therapy, immunosuppressant/immunomodulator therapy, and infliximab or adalimumab/surgery. Because tissue samples were obtained during colonoscopy and the samples were stored until they were processed for DNA isolation, physicians determined whether to administer anti-CMV therapy or not based on the results of CMV-antigenemia or -IHC in the biopsy specimens. Therefore, in this study, the clinical course was independent of the results of CMV-DNA. Finally, we evaluated the UC clinical course between CMV-DNA− cases and CMV-DNA+ cases with low viral load. We compared nominal variables or continuous variables between both groups using the χ2 test, Fisher’s exact, or Mann-Whitney U tests, as appropriate. Values of *p*<0.05 were considered significant. All statistical analyses were performed using Stata software (Version14, Stata Co, College Station, TX).

## Results

### Baseline characteristics

The baseline clinical characteristics of the 46 patients with UC are shown in Table 1. Median age was 44 years and 26 (56%) of the patients were male. Median disease activity index score was 8.5. The involvement distribution comprised left-sided colitis in 39% and extensive colitis in 61%. Thirty-seven patients (80%) had been treated with 5-ASA, 17 (37%) with corticosteroids, 9 (19%) with azathioprine, 1 (2%) with apheresis, 2 (4%) with tacrolimus, 1 (2%) with cyclosporine, 1 (2%) with infliximab, and none with adalimumab. Median dose of corticosteroids was 0mg/4weeks. CMV antigenemia was assayed in 26 of the 46 patients. Four CMV-DNA+ patients were CMV-antigenemia positive (44%) and 1 CMV-DNA− patient was CMV-antigenemia positive (6%) (Table 1). CMV IHC was evaluated in 29 of the 46 patients. Five CMV-DNA+ patients were CMV-IHC positive (56%) and 1 CMV-DNA− patient was CMV-IHC positive (5%) (Table 1). Moreover, CMV serology testing was performed in 24 of the 46 patients. All 7 CMV-DNA+ patients who were underwent CMV serology testing were CMV-seropositive (100%) and 9 CMV-DNA− patients were CMV-seropositive (53%). The baseline characteristics and clinical course among these 3 groups (CMV-DNA−, CMV-DNA+ with low load, and with high load) are shown in S1 Table.

**Table 1:** Baseline characteristics in CMV-DNA+ and CMV-DNA− patients (N = 46).

Characteristics	All (N = 46)	CMV-DNA (+) (n = 12)	CMV-DNA (-) (n = 34)	*p* value
Age (years), median (range)	44 (16–91)	66 (23–91)	39 (16–74)	**0.009**
Sex (male)	26 (56%)	8 (75%)	18 (53%)	0.509
Duration of disease, < 1 year	18 (39%)	3 (25%)	15 (44%)	0.315
Duration of disease, 1–5 years	9 (20%)	3 (25%)	6 (18%)	0.678
Duration of disease, > 5 years	17 (37%)	6 (50%)	11 (32%)	0.314
Duration of disease, Unknown	2 (4%)	0	2 (6%)	1.000
Disease activity index, median (range)	8.5 (2–12)	9 (5–12)	8 (2–12)	0.336
Stool frequency	2.5 (0–3)	3 (1–3)	2 (0–3)	0.184
Rectal bleeding	1 (0–3)	1 (0–3)	1.5 (0–3)	0.804
Endoscopic findings	2.5 (1–3)	3 (2–3)	2 (1–3)	**0.032**
Physician global assessment	2 (0–3)	2 (1–3)	2 (0–3)	0.861
Extensive colitis/ Left-sided colitis	28 (61%)/ 18 (39%)	9 (75%)/ 3 (25%)	19 (56%)/ 15 (44%)	0.315
WBC (10^3^/μl)	7.7 (3.9–17.1)	7.5 (3.9–16.0)	7.7 (3.9–17.1)	0.980
Hb (g/dl)	13.0 (7.5–16.3)	13.4 (9.0–16.3)	12.9 (7.5–16.0)	0.754
Platelet (10^4^/μl)	29.5 (11.0–53.2)	28.3 (18.0–46.5)	30.9 (11.0–53.2)	0.783
Albumin (g/dl)	3.6 (1.7–4.6)	3.4 (1.8–3.9)	3.8 (1.7–4.6)	0.302
CRP (mg/dl)	1.1 (0.2–24.1)	1.5 (0.3–7.4)	0.8 (0.2–24.1)	0.744
ESR (mm/h)	29.0 (2–109)	32.0 (4.0–85.0)	29.1 (2–109)	0.776
CMV antigenemia positive patients ^a^	5 (19%)	4 (44%)	1 (6%)	**0.034**
IHC positive patients ^b^	6 (21%)	5 (56%)	1 (51%)	**0.005**
5-ASA use	37 (80%)	10 (83%)	27 (79%)	1.000
Corticosteroid use	17 (37%)	7 (58%)	10 (29%)	0.093
Dose of Corticosteroids/4w	0 (0–1405)	140 (0–1200)	0 (0–1405)	**0.046**
Azathioprine use	9 (19%)	1 (8%)	7 (21%)	0.660
Apheresis use	1 (2%)	0	1 (3%)	1.000
Tacrolimus use	2 (4%)	0	1 (3%)	1.000
Cyclosporine A use	1 (2%)	0	1 (3%)	1.000
Infliximab use	1 (2%)	0	1 (3%)	1.000

^a^Twenty-six patients underwent CMV antigenemia assay.

^b^Twenty-nine patient sample were subjected to IHC analysis. We compared nominal variables or continuous variables between both groups using the χ2 test, Fisher’s exact, or Mann-Whitney U tests, as appropriate. Bold value means statistical significance.

Abbreviations: NA, not applicable; WBC, white blood cell; Hb, hemoglobin; ESR, erythrocyte sedimentation rate; CRP, C-reactive protein; IHC, immunohistochemistry; 5-ASA, 5-aminosalicylic acid.

### Baseline characteristics associated with CMV-DNA+ patients

A total of 12 patients (27%) were CMV-DNA+ and 34 (73%) were CMV-DNA− (Table 1). CMV-DNA+ patients were significantly older and had a significantly higher endoscopic score than those who were CMV-DNA-. CMV-DNA+ patients had significantly higher rates of CMV-antigenemia positivity and immunohistochemical positivity than the CMV-DNA− patients. The dose of corticosteroids during the past 4 weeks was higher in CMV-DNA+ patients than in CMV-DNA− patients at the beginning of the treatment. No significant differences were observed in other clinical factors between the CMV-DNA+ and CMV-DNA− patients.

### Baseline characteristics and clinical course of CMV-DNA+ patients with and without anti-CMV therapy

Table 2 shows baseline characteristics and the clinical course in the anti-CMV therapy-treated group (cases 1–6) and -untreated group (cases 7–12). Based on the results of CMV-antigenemia or CMV-IHC, we decided whether to administer anti-CMV therapy or not, because the CMVDNA value was not obtained when anti-CMV therapy was initiated. CMV-DNA values were higher in cases 1–6 (high-load CMV-DNA group: median, 16,000; range, 9,000–36,400) than in cases 7–12 (low-load CMV-DNA group: median, 918.5; range, 157–5,480).

**Table 2:** Baseline characteristics and clinical course in CMV-DNA+ patients with and without anti-CMV therapy (n = 12).

Case	UC characteristics at baseline				CMV characteristics				UC clinical course after antivirus therapy						
	DAI score	Steroid use (mg/ 4w)	Steroid refractory/ dependent	Immunomodulator ^a^ use	CMV- Ag*	CMV-IHC**	CMV- DNA	CMV therapy	5-ASA	Steroid	AZA	TAC	CyA	IFX	Outcome
1	5	975	Dependent	AZA	1	0	9,000	Val GCV	→	↘	−	+	−	−	Remission
2	12	845	Dependent		0	4	10,000	GCV/ Foscarnet	→	↘	−	−	−	−	Remission
3	9	200	Dependent		0	4	13,400	Val GCV	→	↘	−	−	−	−	Remission
4	12	880	Refractory		1	0	18,600	GCV	→	↘	+	+	+	−	Remission
5	12	0			11	12	28,500	Val GCV	→	↑↘	−	−	−	−	Remission
6	10	1,200	Dependent		1	13	36,400	GCV	→	↘	+	−	−	+	Surgery
7	8	0			NA	0	157	-	→	-	−	−	−	−	Remission
8	9	0			0	0	200	-	→	↑↘	+	−	−	−	Remission
9	10	260	Refractory		0	0	307	-	→	↑↘	+	−	−	−	Remission
10	8	0			0	0	1,530	-	→	↑↘	−	−	−	−	Remission
11	6	0			NA	0	4,290	-	→	↑↘	−	−	−	−	Remission
12	6	80			NA	0	5,480	-	→	↑↘	+	−	−	−	Remission

^a^immunomodulators include azathioprine, tacrolimus, and cyclosporine.

*number of positive cells,

**number of positive cells.

Arrows indicate changes in medication dose:“→”, no change in dose;“↑↘”, dose was increased then decreased;“↘”, dose was decreased. Abbreviations: NA, not applicable; AZA, azathioprine; Ag, antigenemia; IHC, immunohistochemistry; PCR, polymerase chain reaction; 5-ASA, 5-aminosalicylic acid; TAC, tacrolimus; CyA, cyclosporine; IFX, infliximab; ValGCV, valganciclovir; GCV, ganciclovir.

At baseline before CMV therapy, in the high-load CMV-DNA group (n = 6), 5 used steroid with a mean dose of 683 mg/4weeks, 5 were steroid refractory or dependent, and 1 used azathioprine. In contrast, in the low-load CMV-DNA group (n = 6), 2 used steroid with a mean dose of 170 mg/4weeks, 1 was steroid refractory, and none used immunomodulators. CMV therapy regimens included GCV (n = 2), Val GCV (n = 3), and GCV/Foscarnet (n = 1).

In terms of the clinical course of UC, in the high-load CMV-DNA group (n = 6), 3 patients (cases 2, 3, and 5) achieved remission by antiviral therapy without additional UC therapy, 2 patients (cases 1 and 4) required additional UC therapy (tacrolimus [case1], azathioprine, tacrolimus, and cyclosporine [case 4]) to achieve remission, and the remaining patient (case 6) required colectomy despite receiving azathioprine and infliximab therapy. On the contrary, in the low-load CMV-DNA group (n = 6), all patients (cases 7–12) had remission with additional UC therapy alone, without the need for antiviral therapy.

### Differences in clinical course between CMV-DNA− cases and CMV-DNA+ cases with low viral load

As shown in Table 3, there were no significant differences in UC clinical course among three groups (CMV-DNA−, CMV-DNA+ with low load, and CMV-DNA+ with high load).

**Table 3:** Differences in clinical course among CMV-DNA− patients, CMV-DNA+ patients with low viral load, and CMV-DNA+ patients with high viral load (N = 46).

Characteristics	CMV-DNA(−) (n = 34)	CMV-DNA(+) low viral load (n = 6)	CMV-DNA(+) high viral load (n = 6)	*p* value DNA(−) vs DNA(+) low	*p* value DNA(−) vs DNA(+) high	*p* value DNA(+) low vs DNA(+) high
Response with 5-ASA alone	10 (29%)	1 (17%)	0			
Response with additional PSL alone	14 (41%)	2 (33%)	4 (66%)			
Response with additional PSL and immunosuppressant/ immunomodulator^a^	9 (26%)	3 (50%)	1 (17%)			
Response with additional PSL and immunosuppressant/ immunomodulator and biologics^b^ / surgery	1 (3%)	0 (0%)	1 (17%)	0.683	0.163	0.372

^a^Immunosuppressants/ immunomodulators include azathioprine, tacrolimus, and cyclosporine.

^b^Biologics includes infliximab and adalimumab. The data were analyzed byχ2 test.

Abbreviations: PSL, prednisolone (corticosteroid); Bio, biologics (infliximab, no patients received adalimumab).

## Discussion

In this study, we applied quantitative real-time PCR analysis for the detection of CMV in the colonic mucosa of UC patients. Our three main findings are as follows. First, regarding the patients’ background data, we found that CMV-DNA+ patients were older, had a higher endoscopic score, and required a higher dose of corticosteroids than CMV-DNA− patients. Second, in terms of the clinical course of UC for CMV-DNA+ patients, in the anti-CMV-treated group there were 2 patients who required additional UC therapy and 1 who received colectomy despite receiving azathioprine and infliximab therapy; in the CMV-untreated group, all patients achieved remission with UC therapy alone. Third, no significant difference was observed in the clinical course between CMV-DNA− cases and + cases with low viral loads.

Some reports[5,6,7] have referred to associations of background factors in UC and CMV infection. Consistent with our findings, Matsuoka *et al* evaluated the CMV IgG and antigenemia assay in UC and showed that patients with CMV reactivation were significantly older than those who were CMV IgG negative (40.0 vs 28.0 years old)[7]. Yoshino *et al* evaluated mucosal CMV-DNA in steroid-refractory UC and showed that CMV-DNA+ cases were older than the − cases (44.1 vs 36.5 years old)[6]. In our study, the endoscopic score was higher in CMV-DNA+ than − patients. Suzuki *et al* showed that CMV-positive cases in UC had features of mucosal change such as wide mucosal defects and punched-out lesions[18]. From their studies[6,7,18] and our findings, CMV seems to be more readily identified in severely inflamed colonic mucosa than in mild inflammation as confirmed by endoscopy. In agreement with our study, Sara T et al reported that immunomodulatory agent use such as infliximab was not associated with an increased risk of CMV reactivation[19]. Conversely, we found that the positive relationship between the dose of corticosteroids and CMV-DNA positivity. Some studies have suggested that the prevalence of CMV infection appears to be high in steroid-resistant UC patients[20]. Also, a previous literature review showed that corticosteroid use reduces immunity and increases the risk of gastrointestinal CMV[21]. In addition to CMV, the dose of corticosteroids was significantly associated with fungal gastrointestinal infection[22]. The effect of impaired immunity on CMV reactivation in infliximab use might differ from that of corticosteroid use.

It is not yet known whether CMV worsens colonic inflammation in UC patients, or whether severe UC results in CMV infection that may not exacerbate colonic inflammation. Matsuoka *et al* examined whether or not CMV reactivation should be treated in UC patients receiving immunosuppressive agents and showed that 18 of 25 patients entered remission with conventional immunosuppressive therapies alone without anti-CMV therapy. A study from Fukuchi *et al* showed that there was no significant difference in the proportion of patients who achieved mucosal healing by intensive granulocyte and monocyte adsorptive apheresis regardless of CMV+ and − UC status[4]. In our study, anti-CMV therapy-untreated cases with low-load CMV-DNA (<5,500 copies/μg DNA) had remission with only treatment of UC, in agreement with previous studies. In clinical practice, physicians can be indecisive over whether to administer anti-CMV therapy to patients with UC[23]. UC treatment without anti-CMV therapy may be warranted, particularly in patients with low-load CMV-DNA. However, the method for quantifying CMV-DNA differs among studies[5,6] and CMV-DNA might be diluted with DNA derived from infiltrative immune cells in the presence of severe inflammation. Currently, it is difficult to determine the cut-off value for CMV-DNA and further studies involving more patients are needed.

Roblin *et al*, however, reported that quantitative CMV-DNA could predict resistance to steroid treatment in patients with UC[5]. Also, Yoshino *et al* showed that quantitative CMV-DNA was detected only in the inflamed mucosa and a high remission rate was achieved in UC patients refractory to immunosuppressive therapy by administering either antiviral or immunosuppressive therapy[6]. In agreement with this, 3 patients in our study achieved remission by antiviral therapy without additional UC therapy. Moreover, 1 patient required colectomy despite receiving anti-CMV therapy and additional UC treatment. Therefore, randomized clinical trials are needed in the future to determine whether anti-CMV therapy induces remission or not in patients with high-load CMV-DNA. We need to re-acknowledge that anti-CMV therapy alone does not always achieve clinical response in UC even in high-load DNA cases.

This study has some limitations. First, the number of subjects in our study was small (N = 46), but previous studies evaluating CMV-DNA in patients with UC also involved a small sample size such as Yoshino *et al*[6] and Robin *et al*[5] (N = 30 and N = 42, respectively). Second, the treatment strategy was based on each physician’s decisions and the patients’ preferences because this study was retrospective in nature. Third, our study involved a heterogenous cohort, which might have been different from previous studies[4,5,6] evaluating the clinical course in CMV-DNA+ UC. In contrast, some advantages are that we could collect detailed clinical information on both baseline data and the clinical course by using an electronic medical database. Although the main drawback of colonic tissue PCR is that it is overly sensitive and is postulated to detect mild reactivations of CMV, we recognize the appropriateness of a bellwether for CMV reactivation. Yoshino *et al*[6] defined CMV reactivation as a positive finding on quantitative real-time PCR, as we did in our study. The current ECCO guidelines recommend colonic tissue PCR as the preferred test for screening for CMV colitis[24].

In conclusion, older age, corticosteroid therapy, and active inflammation predispose to CMV infection of the colonic mucosa in UC. The clinical course of UC was not affected in patients with low-load CMV-DNA and no viral therapy; however, half of the patients with high-load CMV-DNA derived some benefit from anti-CMV therapy. These data suggest that UC treatment without anti-CMV therapy may be warranted, particularly in patients with low-load CMV-DNA, whereas anti-CMV therapy alone does not always achieve clinical response in UC even in cases with high-load DNA.

## Supporting information

S1 Table Baseline characteristics among CMV-DNA− patients, CMV-DNA+ patients with low viral load, and CMV-DNA+ patients with high viral load (N = 46). Note: ^a^Twenty-six patients underwent CMV antigenemia. ^b^Twenty-nine patient samples were subjected to IHC analysis. We compared nominal variables or continuous variables between both groups using the χ2 test, Fisher’s exact, or Mann-Whitney U tests, as appropriate. Bold value means statistical significance. Abbreviations: NA, not applicable; WBC, white blood cell; Hb, hemoglobin; ESR, erythrocyte sedimentation rate; CRP, C-reactive protein; IHC, immunohistochemistry; 5-ASA, 5-aminosalicylic acid.
(DOCX)

## Abbreviations

CMV: cytomegalovirus
DNA: deoxyribonucleic acid
PCR: polymerase chain reaction
PSL: prednisolone
UC: ulcerative colitis
