# Supplementary material for: Colonic cytomegalovirus detection by mucosal PCR and antiviral therapy in ulcerative colitis
Source: PLoS One. 2017 Sep 8;12(9):e0183951. doi: 10.1371/journal.pone.0183951 (PMC5590814; doi:10.1371/journal.pone.0183951)
Supplement: S1 Table — Note: aTwenty-six patients underwent CMV antigenemia. bTwenty-nine patient samples were subjected to IHC analysis. We compared nominal variables or continuous variables between both groups using the χ2 test, Fisher’s exact, or Mann-Whitney U tests, as appropriate. Bold value means statistical significance. Abbreviations: NA, not applicable; WBC, white blood cell; Hb, hemoglobin; ESR, erythrocyte sedimentation rate; CRP, C-reactive protein; IHC, immunohistochemistry; 5-ASA, 5-aminosalicylic acid. (DOCX) [file pone.0183951.s001.docx]

**Supplementary Table 1. Baseline characteristics among CMV-DNA− patients, CMV-DNA+ patients with low viral load, and CMV-DNA+ patients with high viral load (N = 46).**

| Characteristics | CMV-DNA(-) (n = 34) | CMV-DNA(+) low viral load (n=6) | CMV-DNA(+) high viral load (n=6) | *p* value DNA(-) vs DNA(+) low | *p* value DNA(-) vs DNA(+) high | *p* value DNA(+) low vs (+) high |
| --- | --- | --- | --- | --- | --- | --- |
| Age (years), median (range) | 39 (16-74) | 55.5 (30-91) | 66.5 (23-69) | 0.116 | **0.017** | 0.931 |
| Sex (male) | 18 (53%) | 4 (67%) | 4 (67%) | 0.673 | 0.673 | 1.000 |
| Duration of disease, < 1 year | 15 (44%) | 2 (33%) | 1 (17%) | 1.000 | 0.373 | 1.000 |
| Duration of disease, 1-5 years | 6 (18%) | 0 | 3 (50%) | 0.565 | 0.115 | 0.182 |
| Duration of disease, > 5 years | 11 (32%) | 4 (67%) | 2 (33%) | 0.174 | 1.000 | 0.567 |
| Duration of disease, unknown | 2 (6%) | 0 | 0 | 1.000 | 1.000 | NA |
| Disease activity index, median (range) | 8.0 (2-12) | 8.0 (6-10) | 11.0 (5-12) | 0.848 | 0.099 | 0.104 |
| Stool frequency | 2.0 (0-3) | 2.5 (1-3) | 3.0 (1-3) | 0.627 | 0.129 | 0.330 |
| Rectal bleeding | 1.5 (0-3) | 1.0 (0-2) | 2.0 (0-3) | 0.797 | 0.523 | 0.560 |
| Endoscopic findings | 2.0 (1-3) | 3.0 (2-3) | 3.0 (2-3) | 0.186 | 0.0569 | 0.520 |
| Physician global assessment | 2.0 (0-3) | 2 (1-2) | 2.0 (2-3) | 0.104 | 0.055 | **0.006** |
| Extensive colitis/ Left-sided colitis | 19 (56%)/ 15 (44%) | 4 (66%)/ 2 (33%) | 5 (83%)/ 1 (17%) | 1.000 | 0.373 | 1.000 |
| WBC (10^3^/µl) | 7.7 (3.8-17.1) | 7.4 (5.9-16.0) | 7.5 (3.95-10.3) | 0.595 | 0.622 | 0.873 |
| Hb (g/dl) | 12.9 (7.5-16) | 13.9 (10.7-16.3) | 11 (9-15.9) | 0.240 | 0.483 | 0.173 |
| Platelet (10^4^/µl) | 30.9 (11-53.2) | 28.9 (23-39.5) | 27.9 (18-46.5) | 0.115 | 0.638 | 0.745 |
| Albumin (g/dl) | 3.8 (1.7-4.6) | 3.6 (3.1-3.9) | 3.1 (1.8-3.6) | 0.718 | 0.212 | 0.079 |
| CRP (mg/dl) | 0.8 (0.2-24.1) | 1.5 (0.3-7.4) | 1.5 (0.5-6.0) | 0.937 | 0.666 | 0.855 |
| ESR (mm/h) | 29.0 (2-109) | 46 (4-85) | 30.5 (4-53) | 0.822 | 0.850 | 0.468 |
| CMV antigenemia, positive patients ^a^ | 1 (6%) | 0 | 4 (66%) | 0.850 | **0.008** | 0.167 |
| IHC positive patients ^b^ | 1 (51%) | 0 | 5 | 1.000 | **< 0.001** | **0.015** |
| 5-ASA use | 27 (79%) | 4 (66%) | 6 (100%) | 0.602 | 0.567 | 0.455 |
| Corticosteroid use | 10 (29%) | 2 (33%) | 5 (83%) | 1.000 | **0.021** | 0.242 |
| Dose of Corticosteroids/4w | 0 (0-1450) | 0 (0-260) | 862.5 (0-1200) | **0.047** | **0.002** | **0.031** |
| Azathioprine use | 7 (21%) | 0 | 2 (33%) | 0.567 | 0.602 | 0.455 |
| Apheresis use | 1 (3%) | 0 | 0 | 1.000 | 1.000 | NA |
| Tacrolimus use | 1 (3%) | 0 | 1 (17%) | 1.000 | 0.281 | 1.000 |
| Cyclosporine A use | 1 (3%) | 0 | 0 | 1.000 | 1.000 | NA |
| Infliximab use | 1 (3%) | 0 | 0 | 1.000 | 1.000 | NA |

Note: ^a^Twenty-six patients underwent CMV antigenemia. ^b^Twenty-nine patient samples were subjected to IHC analysis. We compared nominal variables or continuous variables between both groups using the χ2 test, Fisher’s exact, or Mann-Whitney U tests, as appropriate. Bold value means statistical significance.

Abbreviations: NA, not applicable; WBC, white blood cell; Hb, hemoglobin; ESR, erythrocyte sedimentation rate; CRP, C-reactive protein; IHC, immunohistochemistry; 5-ASA, 5-aminosalicylic acid.
